# Supplementary material for: Synthesis of Gold-Platinum Core-Shell Nanoparticles Assembled on a Silica Template and Their Peroxidase Nanozyme Properties
Source: Int J Mol Sci. 2022 Jun 8;23(12):6424. doi: 10.3390/ijms23126424 (PMC9223353; doi:10.3390/ijms23126424)
Supplement: Supplementary file 1 [file ijms-23-06424-s001.zip › ijms-1723342-supplementary.pdf]

Synthesis of gold–platinum core–shell nanoparticles assembled on a silica template and their peroxidase nanozyme properties

Xuan-Hung Pham [1], Van-Khue Tran [2], Eunil Hahm [1], Yoon-Hee Kim [1], Jaehi Kim [1], Wooyeon Kim [1] and Bong-Hyun Jun [1,*]

[1] Department of Bioscience and Biotechnology, Konkuk University, Seoul 05029, Republic of Korea; e-mail: phamricky@gmail.com (X.-H.P.); greenice@konkuk.ac.kr (E.H.); hilite2201@naver.com (Y.-H.K.); susia45@gmail.com (J.K.); buzinga5842@konkuk.ac.kr (W.K.); bjun@konkuk.ac.kr (B.-H.J.)

[2] VNUK Institute for Research and Executive Education, The University of Danang, Danang City, Vietnam; khue.tran@vnuk.edu.vn (V.-K.T.)

* Correspondence: bjun@konkuk.ac.kr; Tel.: +82-2-450-0521.

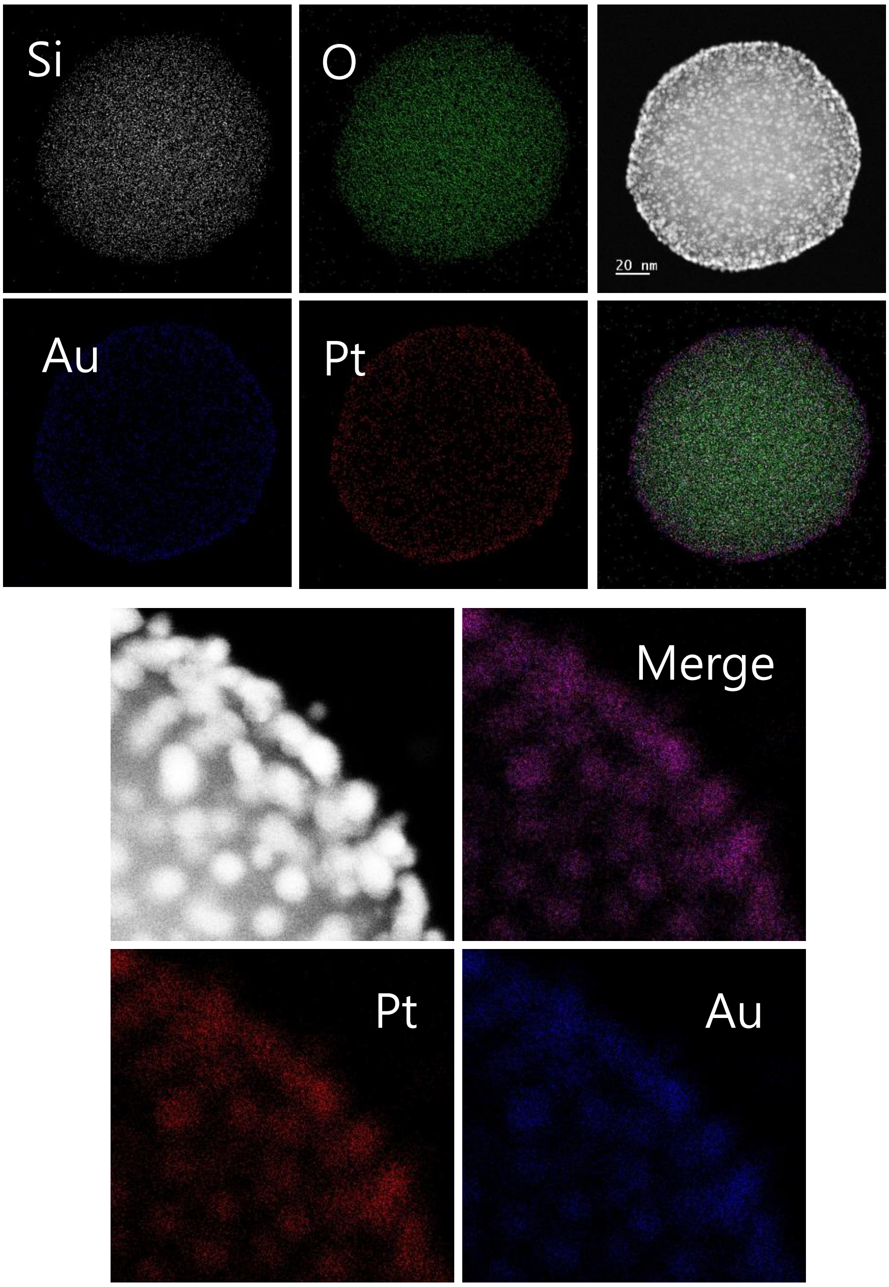

Figure S1. Energy-dispersive X-ray spectroscopy mapping of SiO_2@Au@Pt for the SiO_2, Au, and Pt elements. SiO_2@Au@Pt was synthesized using 200 μg of SiO_2@Au and 200 μM Pt^{2+}.

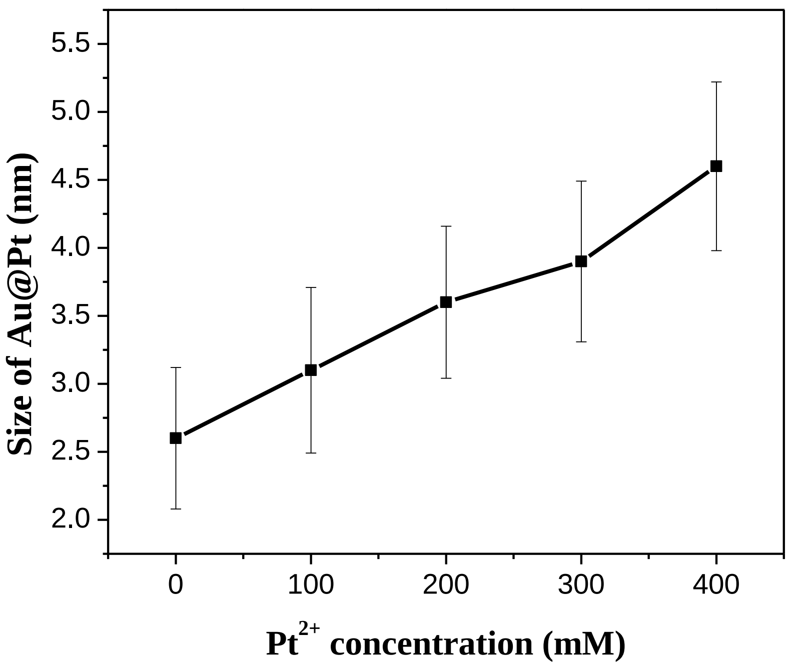

Figure S2. Sizes of Au@Pt on the surface of SiO_2@Au@Pt synthesized using 200 μg of SiO_2@Au and various Pt^{2+} concentrations (from 0 to 400 μM).

Table S1. Effects of various Pt^{2+} concentrations on the reciprocal distribution of Pt and Au on the surface of SiO_2@Au@Pt synthesized using 200 μg of SiO_2@Au and various concentrations of Pt^{2+}.

Pt^{2+} concentration (μM)	Pt	Au	Reciprocal of Pt and Au
0	0	100	0
100	71.59	28.41	2.5
200	76.4	23.6	3.2
300	79.92	20.08	4.0
400	87.07	12.93	6.7

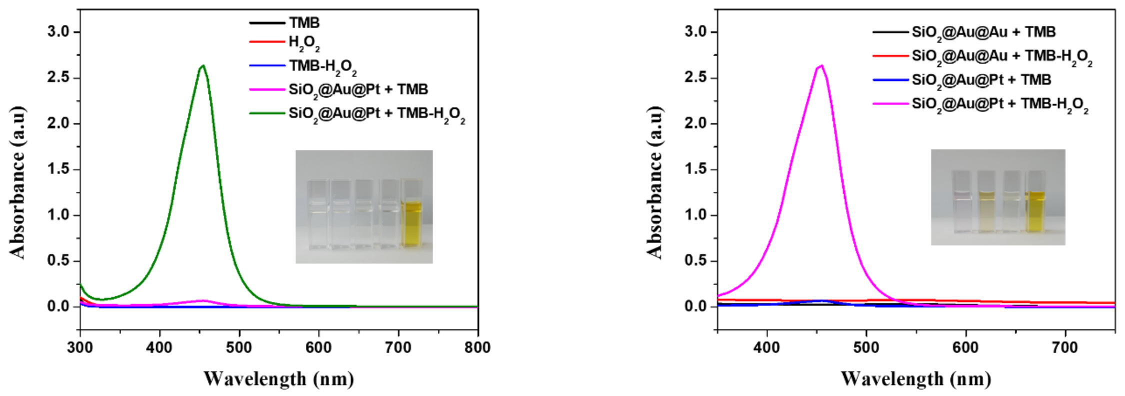

Figure S3. (a) UV–Vis absorbance spectra and an optical image (inset) of SiO_2@Au@Pt (5 μg) in TMB and H_2O_2 solutions. (b) UV–Vis absorbance spectra and an optical image (inset) of SiO_2@Au@Pt (5 μg) and SiO_2@Au@Au (5 μg) in TMB and TMB-H_2O_2 solutions.

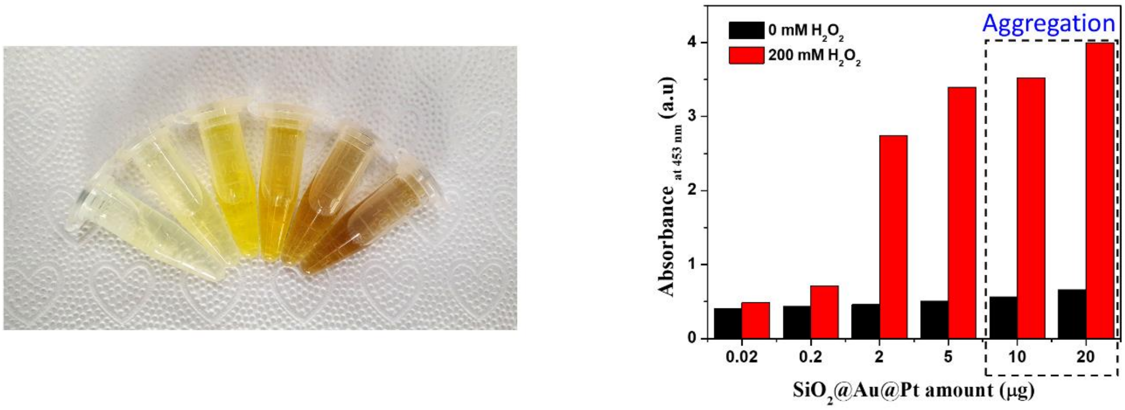

Figure S4. Effects of the amount of SiO_2@Au@Pt NPs (0.02–20 μg) on the peroxidase-like activity of SiO_2@Au@Pt in the presence of 0.6 mM TMB and 200 mM H_2O_2 at pH 4.0 in a 15-min reaction. The UV−Vis absorbance plots at 453 nm for each condition represent the absorbance of the oxidized TMB substrate.

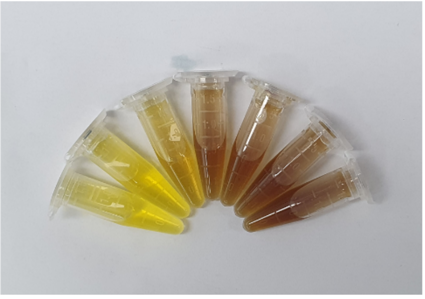

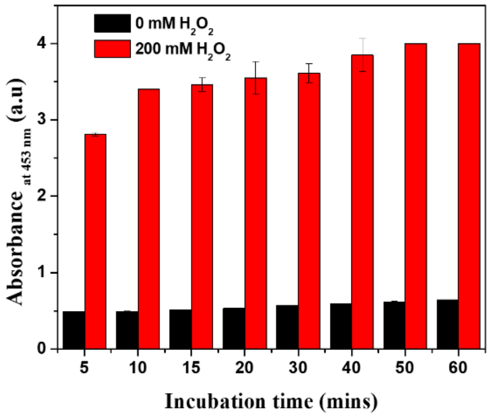

Figure S5. Effects of the incubation time (5–60 min) on the peroxidase-like activity of SiO_2@Au@Pt in the presence of 0.6 mM TMB and 200 mM H_2O_2 at pH 4.0. The UV−Vis absorbance plots of each condition at 453 nm represent the absorbance of the oxidized TMB substrate.

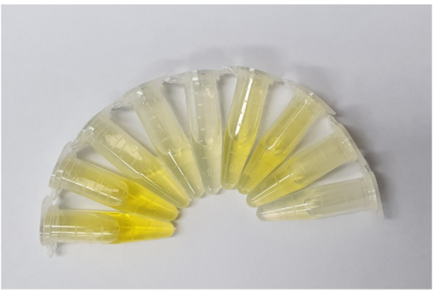

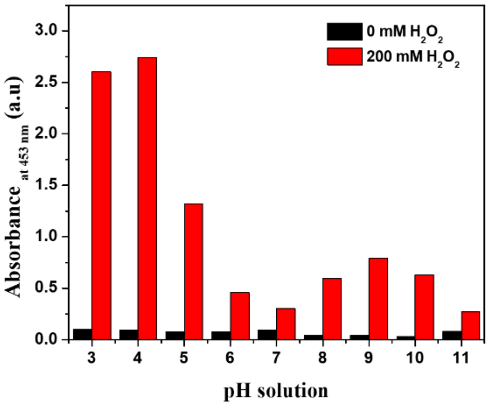

Figure S6. Effects of pH (3.0–11.0) on the peroxidase-like activity of SiO_2@Au@Pt in the presence of 0.6 mM TMB and 200 mM H_2O_2 in a 15-min reaction. The UV–Vis absorbance plots of each condition at 453 nm represent the absorbance of the oxidized TMB substrate.

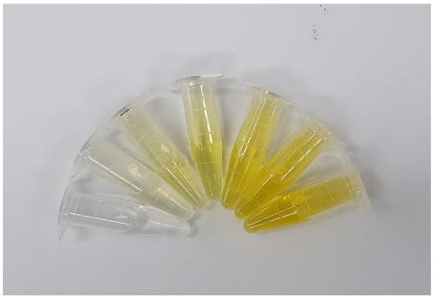

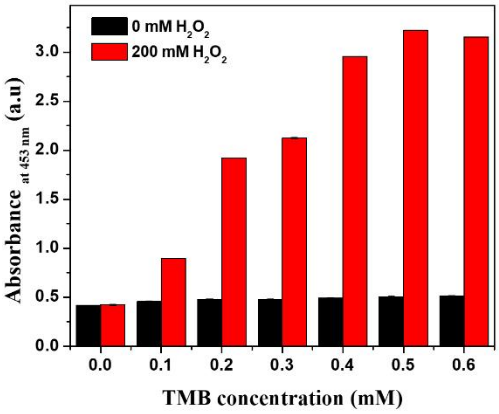

Figure S7. Effects of the TMB concentration (0–0.6 mM) on the peroxidase-like activity of SiO_2@Au@Pt in the presence of 200 mM H_2O_2 at pH 4.0 in a 15-min reaction. The UV–Vis absorbance plots of each condition at 453 nm represent the absorbance of the oxidized TMB substrate.

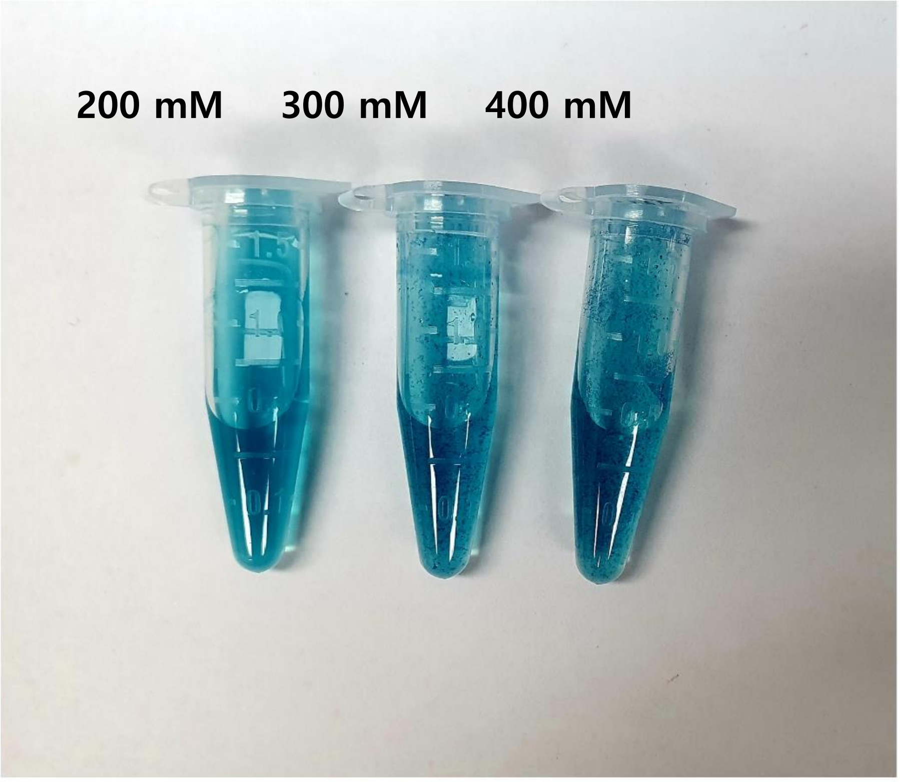

Figure S8. Colors of SiO_2@Au@Pt@Pt NPs with 200, 300, and 400 mM H_2O_2 in the presence of 0.5 mM TMB. The optimized conditions are 5 μg of SiO_2@Au@Pt, 0.6 mM TMB, and pH 4.0 in a 15-min reaction.
